# Editorial: Torpor and hibernation: metabolic and physiological paradigms

**DOI:** 10.3389/fphys.2024.1441872

**Published:** 2024-06-18

**Authors:** Sylvain Giroud, Yoshifumi Yamaguchi, Jeremy Terrien, Robert H. Henning

**Affiliations:** ^1^ Energetics Lab, Department of Biology, Northern Michigan University, Marquette, MI, United States; ^2^ Hibernation Metabolism, Physiology, and Development Group, Institute of Low Temperature Science, Hokkaido University, Sapporo, Japan; ^3^ Graduate School of Environmental Science, Hokkaido University, Sapporo, Japan; ^4^ Unité Mécanismes Adaptatifs et Evolution (MECADEV), Muséum National d’Histoire Naturelle, Centre National de la Recherche Scientifique Unité Mixte de Recherche 7179, Brunoy, France; ^5^ Department of Clinical Pharmacy and Pharmacology, University Medical Center Groningen, Groningen, Netherlands

**Keywords:** heterothermy, seasonality, thermoregulation, body temperature, pathophysiology, mammals, primates, biomimicry (biomimetics)

Torpor or heterothermy manifest a state of depressed metabolism and feature specific metabolic, cellular and molecular adaptations that often are seasonal (Jastroch et al., 2016; Giroud et al., 2021). The exact mechanisms and functioning of these extraordinary adaptations are poorly understood. Yet their unraveling will advance our understanding of the orchestration of hibernation and may inspire research related to obesity and metabolic syndrome (Martin, 2008), cardiovascular and metabolic dysfunctions (Nelson and Robbins, 2015; Bonis et al., 2018), ischemia-reperfusion injuries (Drew et al., 2001; Kurtz et al., 2006), immune depression (Bouma et al., 2010), and longevity of animal species (Keil et al., 2015). Collectively, the Research Topic covers three main aspects on metabolic and physiological changes associated to the phenotype of torpor across several heterothermic species: (i) seasonal metabolic and somatic changes in hibernators; (ii) thermogenic mechanisms, cryoprotection and resistance to metabolic depression; and (iii) mechanisms enabling the induction of a torpid state or “synthetic torpor.”

Hibernators undergo marked seasonal changes in energy metabolism with large differences between an active reproductive season and a period of metabolic depression conveying winter survival. To accommodate seasonal fluctuations, fat-storing hibernators particularly master the circannual cycle of promoting storage or mobilizing lipids. The energy balance of hibernators is regulated by several hormones notably during pre-hibernation fattening (Florant and Healy, 2012). Insulin control of carbohydrate and lipid metabolism is central in regulating cycles of intermittent fasting in mammalian hibernators. Blanco et al. examine glucose and insulin dynamics across the feast-fast cycle in fat-tailed dwarf lemurs (*Cheirogaleus medius*), the only obligate hibernator among primates, showing mechanisms involved in lean-season insulin resistance. In the same vein, Heinis et al. highlight the main metabolic pathways occurring during hibernation by reporting the polar metabolomic profile of white adipose tissue isolated from active and hibernating thirteen-lined ground squirrels (*Ictidomys tridecemlineatus*).

While hibernation interrupts the reproductive cycle in many heterothermic mammals, some hibernating bats engage in mating during hibernation. Sato et al. report males of little horseshoe bats (*Rhinolophus cornutus*) retaining sexual behavior and copulating with females during hibernation. Forced mating appears to increase chances of male bats to obtain a mate while avoiding pre-mating female selection, whereas forced copulations induced arousal in torpid females, which then cannot opt for higher-quality males. The seasonal metabolic changes occurring in hibernators are also associated with changes in individuals’ somatic maintenance such as variations in telomeres, the protective endcap of chromosomes. During hibernation, periodic rewarming, known as interbout arousals, are associated with high metabolic costs including telomeres shortening which can be lengthened in case of extra-energy available during the winter (Giroud et al., 2023) or during the active season (Hoelzl et al., 2016). Galindo-Lalana et al. investigate telomerase activity, a key mechanism in telomere elongation, in the garden dormouse (*Eliomys quercinus*) that shows high telomerase activity across seasons except prior to hibernation due to diversion of resources to increase fat reserves before overwintering.

Besides their seasonal adaptations to overcome challenging conditions, hibernators display powerful metabolic and protective mechanisms, including thermogenesis and cold resistance, to accommodate the physiological extremes and metabolic depression. During arousals, body temperature rapidly rises from 1°C to 40°C requiring tight thermoregulation to maintain rheostasis. Hunstiger et al. reveal differential timing of protein and metabolite abundance of non-shivering thermogenic pathways across different organs in Arctic ground squirrels (*Urocitellus parryii*), indicating distinct thermogenic functions. To extent the understanding of thermoregulatory mechanisms and activation of pro-survival factors during hibernation, Emser et al. studied the mitochondrial single-nucleotide polymorphism m.3017C>T in the evolutionarily conserved gene MT-SHLP6. In-silico analysis indicates the protein truncating polymorphism to be more abundant in heterotherms. Transcript abundance of MT-SHLP6 in thirteen-lined ground squirrel’s brown adipose tissue, a key thermogenic organ, is also high before hibernation and during arousal and low during torpor and after hibernation.

Most mammals adapt thermal physiology to normothermic temperatures with large deviations leading to organ dysfunction and death. Conversely, hibernators resist long-term cold states, a current knowledge which is now summarized by Sone and Yamaguchi. During torpor, hibernators also suppress blood clotting to survive prolonged periods of immobility and decreased blood flow that would otherwise lead to potentially lethal clots. Yet, upon arousal hibernators must quickly restore normal clotting activity to avoid excess bleeding. De Vrij et al. review the mechanisms underlying inhibition of hemostasis in multiple species of hibernating mammals in perspective of medical applications to improve cold preservation of platelets and antithrombotic therapy.

The induction of a torpid state in humans, named “synthetic torpor,” holds large potential for either long distance space travel or treatments of specific medical conditions, and constitutes an active line of research. To identify underlying mechanisms of torpor, the brain is thought to orchestrate various physiological changes within the organism (Drew et al., 2001). The physiological mechanisms facilitating the switch from an active state to a hibernation phenotype remain to be elucidated. The Siberian chipmunk, a food-storing hibernator, activates AMPK, a protein playing a central role in feeding behavior and metabolic regulation in response to starvation. Kamata et al. report phosphorylation of AMPK in brain of hibernating chipmunks and absence of such in the non-hibernating phenotype, corresponding with differences in lifespan. In the same vein, hyperphosphorylated Tau protein is the hallmark of neurodegeneration. Squarcio et al. elucidate the molecular mechanisms underlying reversible hyperphosphorylation of brain Tau protein during a hypothermic state of “synthetic torpor.” Although still far from application in larger non-heterothermic species, such as swine or humans, the overall similarity in Tau and microglia regulation between natural and “synthetic torpor” offers perspective on safe metabolic reduction in non-hibernating species.

Collectively, this Research Topic summarizes key relevant knowledge into understanding the state of hibernation by highlighting various adaptations associated with cryoprotection and resistance to metabolic depression. We hope that this Research Topic will constitute a solid ground for future collaborative and multidisciplinary research efforts toward the understanding of the hibernation phenotype leading to unravel the mechanisms of torpor induction and maintenance in homeotherms for the development of a state of “synthetic torpor” applicable to humans and other non-heterotherms for therapeutic treatments.
